# Goals of Care, Critical Care Utilization and Clinical Outcomes in Obese Patients Admitted under General Medicine

**DOI:** 10.3390/jcm11247267

**Published:** 2022-12-07

**Authors:** Andy K. H. Lim, Greasha K. Rathnasekara, Priyanka Kanumuri, Janith K. Malawaraarachchi, Zheng Song, Claire A. Curtis

**Affiliations:** 1 Department of General Medicine, Monash Health, Clayton, VIC 3168, Australia; 2 Department of Medicine, School of Clinical Sciences, Monash University, Clayton, VIC 3168, Australia

**Keywords:** obesity, body mass index, general medicine, internal medicine, goals of care, advanced care plan, intensive care, critical care, COVID-19, mortality

## Abstract

Obesity is associated with long-term morbidity and mortality, but it is unclear if obesity affects goals of care determination and intensive care unit (ICU) resource utilization during hospitalization under a general medicine service. In a cohort of 5113 adult patients admitted under general medicine, 15.3% were obese. Patients with obesity were younger and had a different comorbidity profile than patients who were not obese. In age-adjusted regression analysis, the distribution of goals of care categories for patients with obesity was not different to patients who were not obese (odds ratio for a lower category with more limitations, 0.94; 95% confidence interval [CI]: 0.79–1.12). Patients with obesity were more likely to be directly admitted to ICU from the Emergency Department, require more ICU admissions, and stayed longer in ICU once admitted. Hypercapnic respiratory failure and heart failure were more common in patients with obesity, but they were less likely to receive mechanical ventilation in favor of non-invasive ventilation. The COVID-19 pandemic was associated with 16% higher odds of receiving a lower goals of care category, which was independent of obesity. Overall hospital length of stay was not affected by obesity. Patients with obesity had a crude mortality of 3.8 per 1000 bed-days, and age-adjusted mortality rate ratio of 0.75 (95% CI: 0.49–1.14) compared to patients who were not obese. In conclusion, there was no evidence to suggest biased goals of care determination in patients with obesity despite greater ICU resource utilization.

## 1. Introduction

Obesity is a condition of excess body weight or body fat, which can lead to health complications and reduced life expectancy. In Australia for 2017–2018, 31% of all Australians were obese, which is a significant increase from 19% in 1995 [1]. Worldwide, obesity affected 13% of the world’s population in 2016, and has tripled since 1975 [2].

The goals of care form (GOC) is a resuscitation planning tool used by many Australian hospitals [3]. It is used during hospital admission to formulate limitations on treatment and cardiopulmonary resuscitation (CPR) [4]. The GOC allows patients and their substitute decision makers to voice their preferences based on individual choices and their clinical situation, while clinicians can use the GOC to document individualized decisions on resuscitation efforts. After the arrival of the SARS-CoV-2 (COVID-19) pandemic, there was additional pressure to complete the GOC early, particularly prior to transfer from the Emergency Department to an inpatient unit.

Obesity is associated with higher long-term mortality [5], but the shorter-term outcome in hospitalized patients is less clear. Obesity may affect CPR quality and survival after cardiac arrest, due to challenges such as difficult airway management and vascular access, obesity-hypoventilation, and altered mechanics of ventilation. There may be a perception of futility, as reflected in the title of conference and newspaper articles such as “Should clinicians offer CPR to obese patients?“ [6], “Man too obese for CPR” [7], and “Will obesity bankrupt the United States?” [8]. Such perceptions may affect GOC discussions. The association between obesity and increased length of hospital stay may have changed over time [9], although significant challenges still remain [10,11]. Medical patients with obesity tend to have a greater length of stay than surgical patients with obesity, and hospital length of stay can vary significantly between specialties [11]. With advances in technology and the appreciation of the unique requirements of patients with obesity, healthcare organizations may have improved their bariatric services, and older studies on mortality and length of stay may not reflect contemporary experience. 

We wanted to know if obesity had an effect on GOC discussion and documentation and the closely linked issue of ICU utilization, in patients admitted under general medicine. The aims of this study were to (1) determine differences in GOC between patients with and without obesity, (2) compare intensive care utilization, length of stay and mortality by obesity status, and (3) identify any impact of COVID-19. 

## 2. Methods

### 2.1. Study Design and Setting

This was a retrospective cohort study of incident non-elective patients admitted to the general internal medicine service (August 2018–September 2020) at a single center within a tertiary hospital network in Melbourne, Australia. The first patient with COVID-19 was reported in late January 2020 but the impact of the first wave was only evident from March 2020. Thus, we defined the period 1 March 2020 to 30 September 2020 as ‘post-COVID-19’ and the period prior to that as ‘pre-COVID-19.’ The study center is a 520-bed acute hospital, with over 6000 non-elective general medicine admissions annually and a general medicine bed occupancy of 125–140 beds. The 5 general medicine units were staffed by 5 full-time and 15 sessional consultants, 15 medical registrars, and 18 medical residents. There is a 14-bed medical and surgical ICU on site.

### 2.2. Study Participants

Adult patients (18–100 years) acutely admitted under general medicine were identified from the main hospital database. Patients directly admitted under the Hospital-In-the-Home (home care) program from the Emergency Department and patients with a length of stay <2 days (a minimum overnight admission required) were not eligible. We used a computer algorithm to randomly select 200 patients from each calendar month of the study period (all patients were included if <200 patients were available). We excluded patients who discharged against medical advice or transferred to other sites.

### 2.3. Outcomes

The GOC has 4 main categories or levels (Supplementary Figure S1), which are summarized in Table 1. In addition to these categories, the document allows for annotation of specific limits, person involved in discussion, and reasons for limitations. For the primary outcome comparing GOC between patients with and without obesity, we examined (1) distribution of GOC categories, and (2) rates of proper completion. Proper GOC completion was defined as a GOC which was completed ≤2 days of admission and included adequate documentation of discussion (“Reason for” or “Discussed with” section being filled in by patient or substitute decision maker). For the secondary outcomes, we examined ICU utilization, inpatient mortality, and length of stay. For both primary and secondary outcomes, we also examined the impact of the COVID-19 pandemic.

### 2.4. Variable Definitions

Obesity was defined as a body mass index (BMI) ≥30 kg/m^2^, from weight divided by height squared. Weight was determined from nutritional assessment charts, or self-reported. We used the age adjusted Charlson Comorbidity Index (Charlson Index) to collect data on comorbidities. For consistency, chronic kidney disease was defined as per Charlson Index, as a serum creatinine >3 mg/dL (265 µmol/L) or dialysis, which approximates advanced chronic kidney disease based on estimated glomerular filtration rate (eGFR, <30 mL/min/1.73 m^2^) in contemporary classification. 

### 2.5. Statistical Analysis

For descriptive analysis, we report the mean and standard deviation (SD) for normally distributed continuous data, or the median and interquartile range (IQR) for skewed data. For categorical data analysis, we used the chi-squared (χ^2^) or Fisher’s exact test. For group comparisons of continuous data, we used either the *t*-test or Mann–Whitney test, depending on data distribution. We used ordinal logistic regression to determine the association between obesity and GOC as a 4-level ordinal outcome of increasing treatment limitation (category A to D). For parsimony, we allowed for a partial proportional odds model in multivariable analysis [12]. The model was acceptable if the main variable of interest (obesity) met the proportional odds (parallel lines) assumption. We used the negative binomial regression method to analyze length of stay data. Zero-inflated models were not required as patients with a length of stay <2 days were not included in this study. We report the crude risk ratio for inpatient mortality and use a log binomial regression approach to determine the age adjusted risk ratio and 95% confidence interval (CI). Analysis was performed using STATA version 16 (StataCorp, College Station, TX, USA). To account for multiple comparisons in ICU resource utilization, only a *p* < 0.01 was considered statistically significant. A *p* < 0.05 was statistically significant for all other analyses.

## 3. Results

### 3.1. Baseline Characteristics

**Figure 1:**
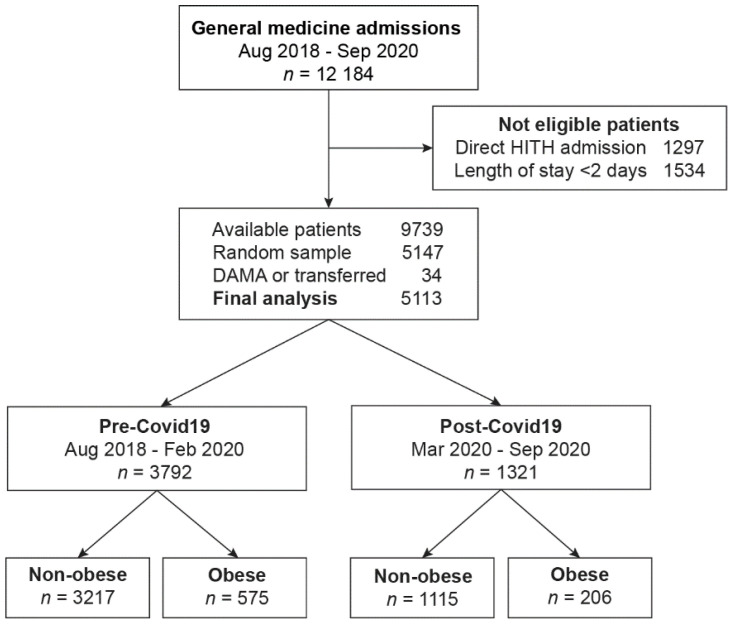
Study flow diagram. DAMA = Discharged against medical advice.

A total of 5113 patients were included in the final analysis (Figure 1), with a total of 37,858 bed-days. There were 781 of 5113 (15.3%) who met the criterion for obesity, and there was no difference in obesity prevalence in the pre- and post-COVID-19 era (15.2% vs. 15.6%, *p* = 0.71). Baseline characteristics of patients are summarized in Table 2. Compared to patients without obesity, patients with obesity were younger but had a greater number of comorbidities. Due to the overall age difference, the Charlson Index was similar in both groups. Patients with obesity were more likely to have diabetes, chronic kidney disease, liver disease, heart failure, peripheral vascular disease, and chronic lung disease. In contrast, patients with obesity were less likely to have a history of transient ischemic attack or stroke, cancer, or dementia. There were fewer patients with obesity who were non-English speaking, but the significance was unclear.

### 3.2. Primary Diagnosis 

A summary of the admission primary diagnosis is shown in Table 3. Infection or sepsis was the most common reason for hospital admission under general medicine, and this was not different between patients with and without obesity (*p* = 0.51). However, patients with obesity were more likely to be admitted with heart failure (*p* < 0.001) or type 2 respiratory failure (*p* = 0.002). On the other hand, patients with obesity were less likely to be admitted with delirium, behavioral disturbance, or psychiatric disorder (*p* = 0.002), and also less likely to be admitted with social or functional decline (*p* = 0.016). 

### 3.3. Obesity and Goals of Care

The admission GOC is summarized in Table 4. Univariable logistic regression analysis is available in Supplementary Table S1. In regressing GOC on obesity, the odds ratio of documenting a lower GOC level (comparing B to A, C to B, or D to C) was 0.66 (95% CI: 0.56–0.77, *p* < 0.001) for patients with obesity compared to patients without obesity. Female sex and non-English speaking status were associated with a lower GOC level, but the effect of female sex was small and insignificant in the multivariable models (thus omitted). Although non-English speaking status was statistically significant in multivariable models, it did not confound the association between obesity and GOC (estimates for obesity changed <2% if omitted). 

A number of comorbidities were associated with GOC, and every 1-point increase in the Charlson Index was associated with 46% higher odds of recording a lower GOC. Dementia was strongly associated with a lower GOC category (odds ratio 4.61, 95% CI: 3.82–5.60), and most clinicians consider dementia in GOC discussions in addition to functional decline (odds ratio 2.37, 95% CI: 1.84–3.05). In the multivariable models (Table 5), the odds ratio for obesity was unchanged after adjusting for functional decline and Charlson Index. However, in models which included age as a covariate (with or without the major morbidities), obesity was not statistically significant. In all models, the proportional odds assumption was tenable (Supplementary Table S2). The proportion of properly completed GOC was lower in patients with obesity compared to patients without obesity (22.7% vs. 26.8%, *p* = 0.017).

### 3.4. Intensive Care Unit Requirement and MET Activation

Compared to patients without obesity, patients with obesity were more likely to be directly admitted to ICU from the Emergency Department, require more ICU admissions overall during hospitalization, and had a greater length of stay in ICU (Table 6). There was no difference in MET activation frequency between the two groups.

Patients with obesity were more likely to be admitted to ICU with respiratory failure (type 2 was more common than type 1 respiratory failure) but less likely to receive intubation and mechanical ventilation. In contrast, patients with obesity were more likely to have non-invasive ventilation compared to patients without obesity. Admission for sepsis, the use of intravenous antibiotics, vasopressors and inotropes, and the incidence of renal replacement therapy were not different between patients with and without obesity. 

### 3.5. COVID-19 and Goals of Care 

When considering the effect of the COVID-19 pandemic, there was a small but statistically significant effect of COVID-19 on GOC. When COVID-19 era was included in the full regression model (Model 4, Table 5), the odds ratio for documenting a lower GOC category was 1.16 (95% CI: 1.01–1.33) in the post-COVID-19 era compared to the pre-COVID-19 era, with other covariates held constant. However, the odds ratio for obesity remained unchanged (−0.5%). An identical result was obtained if the COVID-19 era was included in the simpler model (Model 2, Table 5), with an odds ratio for documenting a lower GOC category of 1.15 (95% CI: 1.01–1.31), with the other covariates held constant. Again, the odds ratio for obesity remained unchanged (−0.3%) even after allowing for the COVID-19 era of treatment.

### 3.6. Inpatient Length of Stay and Mortality

The median hospital length of stay of the cohort was 5 days, and 11.5% of all patients had a length of stay >14 days (Table 7). There were no differences in the hospital length of stay parameters between patients with and without obesity. The incidence of unplanned surgery and interventional radiology procedures were not different between the groups but there were marginally less endoscopic procedures performed on patients with obesity. The overall inpatient mortality was 4.7%, and patients with obesity had 2.1% lower inpatient mortality than patients without obesity (Table 7). 

When considering hospital length of stay, the crude incidence death rate for patients with obesity was 3.8 per 1000 bed-days, compared to 6.8 per 1000 bed-days in patients without obesity (crude incidence rate ratio, 0.55; 95% CI: 0.34–0.85, *p* = 0.004). However, after allowing for the age difference between groups, this was no longer statistically significant (adjusted incidence rate ratio, 0.75; 95% CI: 0.49–1.14, *p* = 0.18). These estimates remain stable even after allowing for COVID-19 era (adjusted incidence rate ratio = 0.75, 95% CI: 0.49–1.15, *p* = 0.19). There was also no statistical interaction between age and obesity (*p* for interaction = 0.35).

## 4. Discussion

In this cohort study of acute multi-day hospitalization, approximately 1 in 7 patients admitted under general internal medicine were obese. Patients with obesity were younger than patients without obesity but demonstrated higher comorbidity burden, particularly diabetes, cardiovascular and chronic lung disease. With regard to admission GOC, the unadjusted estimates showed that patients with obesity had 34% lower odds of documenting a lower category (greater limitations) than patients without obesity. This difference was no longer statistically significant after adjusting for age but remained significant if the Charlson Index was used in the multivariable model instead of age. After the arrival of the COVID-19 pandemic, there was 16% higher odds of documenting a lower GOC category for all patients, without any difference noted for patients with obesity. Patients with obesity demonstrated greater ICU resource utilization in terms of ICU admission rates and length of stay, but obesity was not associated with higher inpatient mortality or total hospital length of stay. Patients with obesity were less likely to receive mechanical ventilation and more likely to receive non-invasive ventilation.

In examining the association between GOC and obesity, we found that age was a significant confounder. Patients with obesity were younger, and every 5-year increase in age was associated with 56% higher unadjusted odds of recording a lower GOC category. Accounting for age in the logistic regression changed the estimates for obesity by around 30%. However, if we used the Charlson Index instead of age, confounding was not evident. This discrepancy may be due to the fact that age per se may be a much stronger determinant of GOC than the Charlson Index in clinical practice. Thus, a younger patient with greater comorbidities may receive a higher GOC level (fewer limitations) compared to an older patient with fewer comorbidities, even if the Charlson Index was similar. This is plausible as patients with obesity had a greater number of comorbidities but scored less points on age compared to patients without obesity, and the end result was a comparable Charlson Index. Furthermore, many of the comorbidities such as heart failure lie on the causal pathway and are theoretically not confounders. We also noted that the proportion of properly completed GOC was lower in patients with obesity, commensurate with the overall higher GOC designation. The observation that patients with higher GOC categories (less limitations) have lower rates of adequate documentation of discussion has been previously reported [13], and presumably reflect the perception that younger or less comorbid patients should default to higher categories (particularly A) without needing discussion.

GOC forms were introduced gradually into Australian hospitals about a decade ago, and one of the aims was to change the culture of medical decision making, by promoting a proactive and timely approach [4]. It is also about sharing prognostic information [14]. Thus, a GOC discussion depends on an accurate assessment of prognosis. The Charlson Index has been around since 1987 [15] and the subsequently modified and validated versions [16,17] have been used for prognostication in many clinical settings, but we are not aware of published papers determining if the Charlson Index is specifically used by clinicians in GOC discussion. We would have expected that if decisions on GOC were based on an assessment of the Charlson Index, there should be no difference in GOC distribution between patients with and without obesity given both groups had similar scores on average. On the other hand, there was very strong evidence that dementia was associated with a lower GOC in univariable analysis. Even though dementia is correlated with age, it was also independently associated with a lower GOC category. Our final interpretation was that the association between obesity and the higher GOC (fewer treatment limitations) was not independent of age. This could represent a decision-making bias, and that certain factors such as dementia and functional decline (both correlating with age) carry more weight in decision making than the sum of comorbidities as assessed with the Charlson Index. 

The effect of obesity on CPR success is complicated. In a large registry study, patients with obesity did not have an increased risk of inpatient mortality after in-hospital arrest, but patients with BMI >35 kg/m^2^ had lower rates of survival following arrest from shockable rhythms [18]. Cardiogenic arrest survivors with significant coronary artery disease who were obese had worse in-hospital mortality and neurological outcomes [19]. Our study findings are consistent with observations in other studies that patients with obesity have greater health care utilization but not increased mortality. In the large French CONSTANCES cohort study, men with obesity had 18% higher odds of attending Emergency Departments (women had 36% higher odds) compared to men and women without obesity, respectively [20]. As observed in our study, patients with obesity were admitted mainly for cardiovascular or respiratory problems. Patients with obesity were also overrepresented in ICU. An Australian ICU study (2012–2014) found that the proportion of patients in ICU with BMI ≥30 kg/m^2^ was 4% to 8% higher than the prevalence of obesity in the age standardized population at the time, but obesity did not affect ICU or in-hospital mortality [21]. In a large Australian and New Zealand multi-center ICU study (2010–2018), patients with BMI ≥35 kg/m^2^ were overrepresented but neither ICU nor in-hospital mortality was increased in patients with obesity. In fact, patients with level I obesity (BMI 30.0–34.9 kg/m^2^) had the lowest mortality which the authors describe as the obesity paradox [22]. An older meta-analysis of 14 ICU studies (2008) noted that obesity was not associated with increased mortality, but patients with obesity had longer duration of ventilation (average 1.48 days more) and ICU length of stay (average 1.08 days more), compared to patients without obesity [23]. 

To our knowledge, there have been no published studies examining the impact of obesity on GOC discussions. Our other strengths include the large cohort sample and the use of multivariable regression to account for confounding. We also collected data on all significant procedures and surgeries to ensure these did not contribute to any differences in hospital length of stay or mortality. The main limitation is the observational design, which does not allow a causality inference. This was also a single-center study, and our hospital is affiliated with a tertiary referral healthcare network. Thus, the generalizability of our results may depend on the size and setting of individual centers and the availability of bariatric equipment and staff trained to use them. Our findings may not necessarily be applicable in smaller, regional or remote settings where ICU is not available. As these patients were primarily admitted for acute medical problems, the results should not be generalized to surgical patients or electively admitted patients. Another limitation is the broad categorization of obesity which was used in this study. We did not specifically determine if patients with level II obesity (BMI 35.0–39.9 kg/m^2^) and level III obesity (BMI ≥ 40.0 kg/m^2^) would yield different associations compared to patients with level I obesity (BMI 30.0–34.9 kg/m^2^). 

In future, it may be useful to conduct a prospective study utilizing an electronically generated Charlson Index calculated from the electronic medical records (EMR) and made available to clinicians at the time of hospital admission for consideration in GOC discussions. Such a system is feasible to set up and may be able to generate a 1-year prognosis estimate [24,25]. This type of study could allow us to confirm if age and specific morbidities such as dementia is more heavily weighted by clinicians compared to the summated score of the Charlson Index. Extending the study to include multiple centers could improve the generalizability, assuming that both EMR and GOC systems are comparable across these centers.

## 5. Conclusions

In contemporary general internal medicine admissions, we did not find evidence that patients with obesity received biased determination of GOC or restriction of access to critical care resources. Patients with obesity have a length of stay and in-hospital mortality rates comparable to patients without obesity.

## Figures and Tables

**Table 1:** Summary of goals of care categories (levels).

Goals of Care Level	General Description
A No limitation	Goal is curative or restorative. Full resuscitation and all life sustaining treatment. For CPR ^1^ and MET ^2^ activations.
B Limitation of treatment	Not for CPR/Code Blue but patient is for MET activation. Limits on vasopressor/inotrope support can be specified.
C Supportive/Palliative	Not for ICU admission, CPR/Code Blue or MET activation. Non-invasive ventilation may be specified if appropriate.
D Terminal	Patient is actively dying, with a prognosis of hours or days. Focus on palliative efforts and symptom management only.

^1^ A Code Blue is called for cardiopulmonary arrest requiring immediate cardiopulmonary resuscitation (CPR). ^2^ A Medical Emergency Team (MET) is activated by deterioration in vital signs or clinical concern. The MET consists of an intensive care unit (ICU) doctor and nurse, general medicine doctor, and support staff with equipment and expertise to recreate ICU care at the bedside in a short period of time. Note: Further details can be entered into a goals of care form beyond category selection shown here; see Supplementary Figure S1.

**Table 2:** Baseline characteristics of general medicine patients by obesity status (*n* = 5113).

Patient Characteristic	Non-Obese *n* = 4332	Obese *n* = 781	*p*-Value
Age, median (IQR), years	74 (60–83)	68 (55–77)	<0.001
Female sex, *n* (%)	2202 (50.8)	417 (53.4)	0.19
Non-English speaking, *n* (%)	998 (23.0)	129 (16.5)	<0.001
Charlson comorbidity index, mean (SD)	5.1 (3.0)	5.0 (2.9)	0.43
Charlson comorbidity index >3, *n* (%)	3046 (70.3)	536 (68.6)	0.34
Number of morbidities, mean (SD)	1.9 (1.5)	3.2 (1.6)	<0.001
Diabetes mellitus, *n* (%)	1305 (30.1)	383 (49.0)	<0.001
Chronic kidney disease, *n* (%)	410 (9.5)	94 (12.0)	0.026
Chronic liver disease, *n* (%)	255 (5.9)	60 (7.7)	0.06
Coronary artery disease, *n* (%)	727 (16.8)	131 (16.8)	0.99
Heart failure, *n* (%)	805 (18.6)	272 (34.8)	<0.001
Stroke/transient ischemic attack, *n* (%)	637 (14.7)	93 (11.9)	0.040
Peripheral vascular disease, *n* (%)	278 (6.4)	76 (9.7)	0.001
Chronic lung disease, *n* (%)	877 (20.2)	205 (26.3)	<0.001
Malignancy, *n* (%)	831 (19.2)	111 (14.2)	0.001
Connective tissue disease, *n* (%)	712 (16.4)	119 (15.2)	0.40
Dementia, *n* (%)	510 (11.8)	43 (5.5)	<0.001
Infection or sepsis, *n* (%)	1375 (31.7)	238 (30.5)	0.49

**Table 3:** Primary diagnosis for medical admission in order of frequency.

Diagnosis Category	Total *N* (%)	Non-Obese *n* (%)	Obese *n* (%)
Infection, sepsis	1610 (31.5)	1372 (31.7)	238 (30.5)
Falls, trauma	462 (9.0)	405 (9.4)	57 (7.3)
COPD, asthma, type 2 resp failure	404 (7.9)	321 (7.4)	83 (10.6)
Heart failure	353 (6.9)	254 (5.9)	99 (12.7)
Cancer-related	295 (5.8)	263 (6.1)	32 (4.1)
Neurological ^1^	284 (5.6)	249 (5.7)	35 (4.5)
Delirium, psychiatric, behavioral	236 (4.6)	217 (5.0)	19 (2.4)
Social, functional	228 (4.5)	206 (4.8)	22 (2.8)
Syncope, arrhythmia	139 (2.7)	116 (2.7)	23 (2.9)
Hepatobiliary, intestinal	120 (2.4)	104 (2.4)	16 (2.1)
AKI or metabolic ^2^	118 (2.3)	105 (2.4)	13 (1.7)
Toxicology	114 (2.2)	102 (2.4)	12 (1.5)
Venous thromboembolism	87 (1.7)	70 (1.6)	17 (2.2)
Acute coronary syndrome	82 (1.6)	70 (1.6)	12 (1.5)
Others	568 (11.1)	468 (10.8)	100 (12.8)

^1^ Stroke, transient ischemic attack, seizures, neuromuscular disease. ^2^ Acute kidney injury, fluid-electrolyte or acid-base disorder, unstable diabetes, ketoacidosis.

**Table 4:** Admission goals of care categories by obesity status (*n* = 5113).

Goals of Care Level	Total *N* (%)	Non-Obese *n* (%)	Obese *n* (%)
Missing	514 (10.1)	433 (10.0)	81 (10.4)
A	2407(47.1)	1983 (45.7)	424 (54.3)
B	1472 (28.8)	1269 (29.3)	203 (26.0)
C	695 (13.6)	623 (14.4)	72 (9.2)
D	25 (0.5)	24 (0.6)	1 (0.1)

**Table 5:** Multivariable ordinal logistic regression of goals of care on obesity status (*n* = 4599).

Model and Variables	Odds Ratio ^1^	95% CI
Model 0: Obesity (univariable)	0.66	0.56–0.77
Model 1: Model 0 + functional decline, Charlson Index	0.65	0.55–0.78
Model 2: Model 0 + functional decline, age	0.94	0.79–1.11
Model 3: Model 2 + dementia	1.00	0.84–1.19
Model 4: Model 3 + heart failure, COPD, CKD, cancer, stroke	0.97	0.80–1.16

Abbreviations: C.I., confidence interval; COPD, chronic obstructive lung disease; CKD, chronic kidney disease. ^1^ Adjusted odds ratio for obesity with covariates held constant.

**Table 6:** Intensive care unit utilization and medical emergency team activation by obesity status.

Characteristic	Total *N* = 5113	Non-Obese *n* = 4332	Obese *n* = 781	*p*-Value
Direct ICU admission, *n* (%)	478 (9.4)	383 (8.8)	95 (12.2)	0.003
Any ICU admission, *n* (%)	611 (12.0)	485 (11.2)	126 (16.1)	<0.001
MET activation, *n* (%)	526 (10.3)	437 (10.1)	89 (11.4)	0.27
≥2 MET activation, *n* (%)	160 (3.1)	137 (3.2)	23 (2.9)	0.75
**Reason for ICU admission**	***N* = 611**	***n* = 485**	***n* = 126**	***p*-value**
Sepsis or septic shock, *n* (%)	146 (23.9)	122 (25.2)	24 (19.1)	0.15
Type 1 respiratory failure, *n* (%) ^1^	82 (13.4)	58 (12.0)	24 (19.1)	0.038
Type 2 respiratory failure, *n* (%) ^2^	110 (18.0)	72 (14.9)	38 (30.2)	<0.001
Cardiac arrest/cardiogenic shock, *n* (%)	35 (5.7)	30 (6.2)	5 (4.0)	0.40
Metabolic support, *n* (%) ^3^	47 (7.7)	43 (8.9)	4 (3.2)	0.037
Hemorrhage, including GIT, *n* (%)	8 (1.3)	7 (1.4)	1 (0.8)	0.99
Trauma, *n* (%)	18 (3.0)	17 (3.5)	1 (0.8)	0.14
Postoperative care, *n* (%)	8 (1.3)	6 (1.2)	2 (1.6)	0.67
Neurological, *n* (%) ^4^	25 (4.1)	21 (4.3)	4 (3.2)	0.80
Toxicology/overdose, *n* (%)	69 (11.3)	61 (12.6)	8 (6.4)	0.06
Others, *n* (%)	63 (10.3)	48 (9.9)	15 (11.9)	N/A
**ICU requirements**	***N* = 611**	***n* = 485**	***n* = 126**	***p*-value**
Length of stay, median (IQR) hours	55 (34–97)	52 (31–94)	63 (42–106)	0.005
Mechanical ventilation, *n* (%)	145 (23.8)	127 (26.3)	18 (14.3)	0.005
Ventilation hours, median (IQR) ^5^	48 (22–106)	48 (20–107)	52 (35–74)	0.73
Non-invasive ventilation, *n* (%)	182 (29.8)	113 (23.3)	69 (54.8)	<0.001
Renal replacement therapy, *n* (%)	39 (6.4)	29 (6.0)	10 (7.9)	0.43
Intravenous antibiotics, *n* (%)	460 (75.3)	362 (74.6)	98 (77.8)	0.47
Inotrope & vasopressor use, *n* (%)	233 (38.1)	181 (37.2)	52 (41.3)	0.41
Norepinephrine	159 (26.0)	123 (25.3)	36 (28.6)	0.46
Metaraminol	90 (14.7)	68 (14.0)	22 (17.5)	0.33
Epinephrine	32 (5.2)	25 (5.1)	7 (5.6)	0.82
Vasopressin	7 (1.1)	5 (1.0)	2 (1.6)	0.64
Milrinone	3 (0.5)	2 (0.4)	1 (0.8)	0.50
Others	4 (0.7)	3 (0.6)	1 (0.8)	N/A

^1^ Hypoxia with normal pCO_2_, such as heart failure, asthma, pneumonia, pulmonary embolism. ^2^ Respiratory acidosis with high pCO_2_, such as chronic obstructive pulmonary disease, obesity-hypoventilation, and other causes of ventilatory failure. ^3^ Severe electrolyte or acid-base disorders, ketoacidosis, acute kidney injury requiring renal replacement therapy. ^4^ Seizures, stroke, coma, brain injury. ^5^ Among patients who were intubated on mechanical ventilation. Abbreviations: ICU, Intensive care unit; MET, Medical emergency team; GIT, Gastrointestinal tract.

**Table 7:** Procedures, inpatient length of stay and mortality by obesity status.

Characteristic	Total *N* = 5113	Non-Obese *n* = 4332	Obese *n* = 781	*p*-Value
Endoscopic procedures, *n* (%)	99 (1.9)	91 (2.1)	8 (1.0)	0.045
Interventional radiology, *n* (%)	159 (3.1)	131 (3.0)	28 (3.6)	0.41
Minor surgery, *n* (%)	69 (1.4)	58 (1.3)	11 (1.4)	0.87
Major surgery, *n* (%)	43 (0.8)	40 (0.9)	3 (0.4)	0.20
LOS, median (IQR), days	5.0 (3.0–8.9)	5.0 (3.0–8.8)	5.2 (3.2–9.4)	0.13
LOS >14 days, *n* (%)	587 (11.5)	494 (11.4)	93 (11.9)	0.68
Inpatient deaths, *n* (%)	239 (4.7)	216 (5.0)	23 (2.9)	0.013
Died in ICU, *n* (%) ^1^	34 (5.6)	29 (6.0)	5 (4.0)	0.38
Deaths pre-COVID 19, *n* (%) ^2^	185 (4.9)	166 (5.2)	19 (3.3)	0.06
Deaths post-COVID 19, *n* (%) ^2^	54 (4.1)	50 (4.5)	4 (1.9)	0.12

Abbreviations: LOS, length of stay; ICU, intensive care unit. ^1^ Represents ICU mortality, and the denominator is the number of patients admitted to ICU. ^2^ Represents inpatient mortality, and the denominator is the number of patients within each study era, respectively (see Figure 1 for details).

## Data Availability

Deidentified data may be available upon reasonable request from the corresponding author, limited to a specific enquiry, pending approval by the Monash Health Research Directorate, and if requested within a reasonable time from publication.

## Supplementary Materials

The following supporting information can be downloaded at: https://www.mdpi.com/article/10.3390/jcm11247267/s1, Figure S1: Monash Health goals of care form, Table S1: Univariable logistic regression models. Table S2: Detailed multivariable regression model with testing of proportional odds assumption.
