# Supplementary material for: Goals of Care, Critical Care Utilization and Clinical Outcomes in Obese Patients Admitted under General Medicine
Source: J Clin Med. 2022 Dec 7;11(24):7267. doi: 10.3390/jcm11247267 (PMC9786344; doi:10.3390/jcm11247267)
Supplement: Supplementary file 1 [file jcm-11-07267-s001.zip › Table S2.pdf]

Journal of
Clinical Medicine

Table S2. Multivariable ordinal logistic regression of goals of care categories on other predictors ($n = 4599$).

**Model and variables**	**Odds ratio**	**95% C.I.**	***p*-value**	***p-value for parallel lines assumption*** [1]
Model 1				0.66
Obesity	0.65	0.55 – 0.78	<0.001	0.63
Functional or social decline	1.91	1.46 – 2.49	<0.001	0.47
Charlson Index, per 1-point increase	1.46	1.43 – 1.50	<0.001	<0.001
Model 2				0.32
Obesity	0.94	0.79 – 1.11	0.50	0.33
Functional or social decline	1.57	1.20 – 2.04	0.001	0.28
Age, per 5-year increase	1.56	1.52 – 1.60	<0.001	<0.001
Model 3				0.39
Obesity	1.00	0.84 – 1.19	0.99	0.49
Functional or social decline	1.35	1.03 – 1.77	0.030	0.35
Age, per 5-year increase	1.52	1.48 – 1.56	<0.001	<0.001
Dementia	3.84	3.19 – 4.61	<0.001	0.25
Model 4				0.35
Obesity	0.97	0.80 – 1.16	0.71	0.64
Functional or social decline	1.51	1.15 – 2.00	0.003	0.62
Age, per 5-year increase	1.52	1.48 – 1.57	<0.001	<0.001
Dementia	4.61	3.81 – 5.58	<0.001	0.09
Heart failure	1.79	1.53 – 2.07	<0.001	<0.001
Chronic lung disease	1.52	1.31 – 1.75	<0.001	<0.001
Chronic kidney disease	1.35	1.11 – 1.64	0.002	0.24
Cancer	1.40	1.34 – 1.45	<0.001	0.03
Stroke or transient ischemic attack	1.74	1.47 – 2.06	<0.001	0.64

[1] Insignificant test statistic indicates that the final model does not violate the proportional odds (parallel lines) assumption. Test of partial proportional odds are also provided next to each variable in the model.
