# Supplementary material for: Goals of Care, Critical Care Utilization and Clinical Outcomes in Obese Patients Admitted under General Medicine
Source: J Clin Med. 2022 Dec 7;11(24):7267. doi: 10.3390/jcm11247267 (PMC9786344; doi:10.3390/jcm11247267)
Supplement: Supplementary file 1 [file jcm-11-07267-s001.zip › Figure S1 (2).pdf]

Journal of *Clinical Medicine*

MDPI

Supplementary Figure S1.

Electronic version of the goals of care form available on the electronic medical records (EMR) system. The form combines tick boxes and free text fields. Prior to September 2019, a paper version of the goals of care form was in use which was identical to the electronic version.

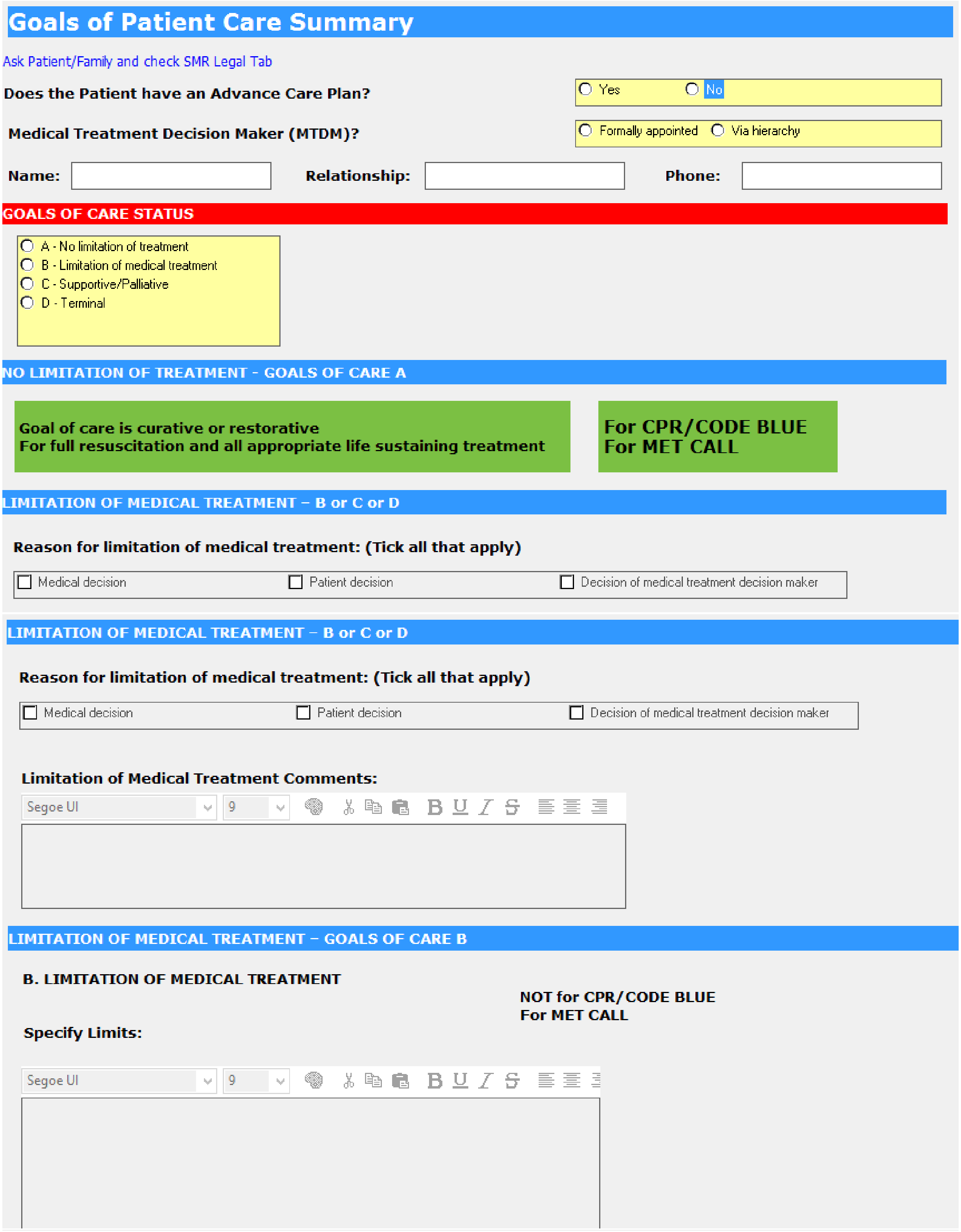

Goals of Patient Care Summary

Ask Patient/Family and check SMR Legal Tab

Does the Patient have an Advance Care Plan? ○ Yes ○ No

Medical Treatment Decision Maker (MTDM)? ○ Formally appointed ○ Via hierarchy

Name: ______ **Relationship:** ______ **Phone:** ______

GOALS OF CARE STATUS

- ○ A - No limitation of treatment
- ○ B - Limitation of medical treatment
- ○ C - Supportive/Palliative
- ○ D - Terminal

NO LIMITATION OF TREATMENT - GOALS OF CARE A

Goal of care is curative or restorative
For full resuscitation and all appropriate life sustaining treatment

For CPR/CODE BLUE
For MET CALL

LIMITATION OF MEDICAL TREATMENT – B or C or D

Reason for limitation of medical treatment: (Tick all that apply)

☐ Medical decision ☐ Patient decision ☐ Decision of medical treatment decision maker

LIMITATION OF MEDICAL TREATMENT – B or C or D

Reason for limitation of medical treatment: (Tick all that apply)

☐ Medical decision ☐ Patient decision ☐ Decision of medical treatment decision maker

Limitation of Medical Treatment Comments:

Segoe UI 9

LIMITATION OF MEDICAL TREATMENT – GOALS OF CARE B

B. LIMITATION OF MEDICAL TREATMENT

NOT for CPR/CODE BLUE
For MET CALL

Specify Limits:

Segoe UI 9

Journal of *Clinical Medicine*

MDPI

LIMITATION OF MEDICAL TREATMENT – GOALS OF CARE C

C. SUPPORTIVE / PALLIATIVE

NOT for ICU ADMISSION
NOT for CPR/CODE BLUE
NOT for MET CALL

Specify Limits:

Segoe UI | 9

For MET CALL:

○ No

LIMITATION OF MEDICAL TREATMENT – GOALS OF CARE D

D. TERMINAL (Prognosis hours or days)

NOT for CPR/CODE BLUE
NOT for ICU ADMISSION
NOT for MET CALL

Specify Limits:

Segoe UI | 9

Start PICD

○ Yes
○ No

Goals of care discussed with:

☐ Patient
☐ Medical Treatment Decision Maker
☐ Previously discussed
☐ Not discussed
☐ Other:

Details of discussion can be found in progress notes on these dates:

Reason not discussed

Doctor Name

Pager:

Designation: ○ Registrar ○ Consultant ○ HMO (in consultation with Registrar/Consultant)

Consultant Medical Practitioner responsible for decision:
